# Demographic Inference of Divergence and Gene Exchange Between *Castanopsis fabri* and *Castanopsis lamontii*

**DOI:** 10.3389/fpls.2020.00198

**Published:** 2020-03-05

**Authors:** Ye Sun, Xiangying Wen

**Affiliations:** ^1^Guangdong Key Laboratory for Innovative Development and Utilization of Forest Plant Germplasm, College of Forestry and Landscape Architecture, South China Agriculture University, Guangzhou, China; ^2^China Office of the Botanic Gardens Conservation International, South China Botanical Garden, Chinese Academy of Sciences, Guangzhou, China

**Keywords:** *Castanopsis*, divergence scenario, gene exchange, niche, restriction site-associated DNA, secondary contact

## Abstract

The cytoplasmic genome of one species may be replaced by that of another species without leaving any trace of past hybridization in its nuclear genome, which can thus confuse the inference of genealogical relationship and evolutionary history of many congeneric species. In this study, we used sequence variations of chloroplast DNA and restriction site-associated DNA to investigate gene exchange between *Castanopsis fabri* and *Castanopsis lamontii*, and to infer the divergence history of the two species by comparing different divergence models based on the joint allele frequency spectrum. We evaluated climatic niche similarity of the two species using climatic variables across their entire distribution range in subtropical China. Clear genetic differentiation was revealed between *C. fabri* and *C. lamontii*, and gene exchange between the two species was discovered as a consequence of secondary contact. The gene exchange rates were variable across the genome. Gene exchange could allow *C. fabri* to widen its habitat through pollen swamping and broaden its climatic niche, and the chloroplast genome of *C. lamontii* is captured by *C. fabri* during this process. These results further our understanding of the timing and contribution of gene exchange to species divergence in forests.

## Introduction

Species that are not completely reproductively isolated can hybridize and form hybrid swarms, with the majority of individuals exhibiting intermediate morphologies and/or mixed genetic characteristics (Milne and Abbott, 2008). However, individuals intermediate in appearance are generally scarce in mixed populations of hybridizing tree species (Whittemore and Schaal, 1991), and introgression is difficult to detect based on morphological characters since backcross hybrids often closely resemble the parental species (Martinsen et al., 2001; Curtu et al., 2007). Gene flow can occur across species boundaries if hybrids backcross to the parental species; as a result, interspecific gene flow is commonly reported among dominant tree species, such as poplars, oaks, and eucalypts (Martinsen et al., 2001; Petit et al., 2003; Mckinnon et al., 2010; Ortego et al., 2018).

Gene exchange can occur over a more widespread area and contribute to geographic expansion in contact zones (Petit et al., 2003; Choler et al., 2004; Khimoun et al., 2011). A large body of studies have revealed that congeneric tree species share chloroplast DNA (Belahbib et al., 2001; Acosta and Premoli, 2009), which have generally been interpreted as consequences of introgressive hybridization (Lexer et al., 2006; Acosta and Premoli, 2009), rather than shared ancestral polymorphism (Muir and Schlötterer, 2005). More extensive chloroplast exchange than nuclear gene exchange are often revealed in plant genera (Rieseberg et al., 1991; Mckinnon et al., 2010). Historical introgression may result in the cytoplasmic genome of one species being replaced by that of another species without leaving any trace of hybridization in its nuclear genome; thus introgression may only be detectable using cytoplasmic marker in these cases (Liu et al., 2010).

Species boundaries are semi-permeable to gene flow as an outcome of competing effects of gene flow and selection against immigrants and/or hybrids (Wu, 2001; De La Torre et al., 2014; Sun et al., 2016). Hybrids selectively filter gene exchange between species, and species identities can be maintained in the face of the interspecific gene flow (Martinsen et al., 2001). Despite recent documentation of differential introgression across the genome, little is known about the demographic history associated with divergence and gene exchange. Specifically accounting for demographic history is necessary to distinguish secondary contact from primary divergence with gene flow (Filatov et al., 2016).

*Castanopsis* is a tree genus of about 120 species mainly distributed in tropical and subtropical regions of Asia. Some taxonomic problems such as high phenotypic variation within widespread species and morphological intergradation between species occur in this genus similar to more intensively studied genus *Quercus*, in which oaks maintain species integrity despite frequent interspecific gene flow (Gailing and Curtu, 2014). *Castanopsis fabri* Hance and *Castanopsis lamontii* Hance are two dominant tree species in evergreen broadleaved forests of subtropical China. *C. fabri* and *C. lamontii* have very similar geographical distribution throughout southeastern China. The two species are closely related, but they are easily distinguished morphologically across their range. *C. fabri* has narrowly oblong or lanceolate leaves with dense epidermal hairs, while *C. lamontii* has glabrous and broadly elliptic leaves. Although co-occurring in forests, *C. fabri* often grows at higher elevation than that of *C. lamontii*, and the latter prefers more moist habitats. The two species grow together in a natural forest stand at Julianshan on the east edge of Nanling mountain region in southeastern China, where the leaf morphology of the two species become blurred in few individuals, potentially as a result of interspecific gene exchange. In this study, we investigate whether gene exchange occurred between these two co-occurring tree species, and infer demographic history of divergence and gene exchange between the two species based on sequence variations of chloroplast DNA and restriction site-associated DNA.

## Materials and Methods

### Sampling and DNA Isolation

Samples were collected from three forest stands at Jiulianshan (coordinates: 24°32′N, 114°27′E), Chebaling (24°43′N, 114°15′E), and Gutian (25°16′N, 116°50′E) on the east edge of Nanling mountain region in southeastern China (Table 1). *C. fabri* and *C. lamontii* occupy different elevations at Chebaling and Gutian, but co-occur at Jiulianshan. We coded individuals of *C. fabri* from Chebaling, Gutian, and Jiulianshan as CB-LF, GT-LF, and JL-LF, and *C. lamontii* from Chebaling, Gutian, and Jiulianshan as CB-LJ, GT-LJ, and JL-LJ. A total of 94 individuals were sampled. Total DNA was isolated with DNeasy Plant Mini Kit (Qiagen, Hilden, Germany) according to manufacturer's instruction.

**Table 1:** Location, code, sample size, and chloroplast haplotype distribution of populations sampled in this study.

**Species**	**Location**	**Population code**	**Individuals**	**Haplotype code and frequency**
*Castanopsis fabri*	Chebaling	CB-LF	10	H4 (10)
	Gutian	GT-LF	10	H3 (3); H4 (7)
	Jiulianshan	JL-LF	42	H2 (32); H4 (10)
*Castanopsis lamontii*	Chebaling	CB-LJ	7	H2 (7)
	Gutian	GT-LJ	8	H2 (8)
	Jiulianshan	JL-LJ	17	H1 (17)

### Chloroplast DNA Sequencing and Analysis

Two chloroplast DNA regions, trnH-psbA, and trnV-trnM (NCBI accession No. MF592798-MF592985), were sequenced in all samples and one individual of *Castanea mollissima* Bl. were used as outgroup. Polymerase chain reaction (PCR) and sequencing were performed with primers described in Kress et al. (2005) and Cheng et al. (2005). A Neighbor-joining tree was constructed using combined chloroplast DNA sequences with MEGA 5.05 (Tamura et al., 2011). Phylogenetic relationships and divergence times among chloroplast haplotype lineages were estimated using Bayesian MCMC (Markov Chain Monte Carlo) algorithm implemented in BEAST v1.8.0 (Drummond and Rambaut, 2007) by including extra outgroups of *Quercus variabilis* Bl. (NCBI accession Nos. KT378288 and KT378292) and *Q. acutissima* Carruth. (NCBI accession Nos. KT378289 and KT378293). A substitution model of GTR+I+G was chosen based on Akaike information criterion (AIC) results from MODELTEST v3.7 (Posada and Crandall, 1998). The Markov chain was started from a random tree and run up to 80,000,000 generations with sampling every 100th cycle. The Markov chain was considered to have reached stationarity when all of the parameters obtained an ESS (effective sample sizes) value of more than 200 as measured in the TRACER 1.6 (Rambaut and Drummond, 2007). The first 200,000 trees were discarded as burn-in, and the remaining data were used to generate a majority-rule consensus tree. Divergence time between the haplotypes was estimated using a Yule tree prior with an uncorrelated lognormal relaxed clock. Two calibration points were chosen based on fossil records and recent phylogenetic studies. First, the earliest unequivocal megafossil of subfamily Castaneoideae of Fagaceae (Crepet and Nixon, 1989) was used to set the root age to 53.50 Ma (normal prior, *SD* = 3 Ma) for all members of *Castanopsis, Castanea*, and *Quercus*. Second, the crown age of *Quercus* was constrained to 35.89 Ma (normal prior, *SD* = 2 Ma) using the most recent phylogenetic study of oaks (Deng et al., 2018).

### Restriction Site-Associated DNA (RAD)-Sequencing and Single Nucleotide Polymorphism (SNP)-Calling

RAD libraries were prepared and sequenced by Floragenex, Inc. (Eugene, Oregon, USA). Briefly, genomic DNA was normalized and digested with restriction endonuclease PstI, then processed into multiplexed RAD libraries following the methods described in Baird et al. (2008). The resulting RAD libraries were sequenced on Illumina (San Diego, CA, USA) HiSeq 2000 platform with single-end 100 bp chemistry. Raw sequenced reads were de-multiplexed and only those reads with sufficiently high sequencing quality (Phred score ≥20) and the correct barcode were retained, then sequencing adaptors and barcodes were removed, and the trimmed RAD fragments were aligned. The Chinese chestnut genome (https://www.hardwoodgenomics.org/bio_data/21) version 1.0 was used as reference to align the de-multiplexed reads with BOWTIE 1.1.1 (Langmead et al., 2009). SNP-calling was performed using SAMTOOLS 0.1.16 (Li et al., 2009), alignment thresholds for the minimum of individual sequencing depth, individual genotype quality, minor allele frequency, and percentage population genotyped, were specified with 6, 10, 0.20, and 75, respectively. A maximum of two mismatches per alignment were permitted to increase alignment stringency and mitigate the effect of possible genome duplication. This had obvious effect of decreasing the number of loci with large numbers of variations present. In the following analyses, one SNP was randomly chosen per RAD locus. It's assumed that our loci are unlinked by keeping only a single SNP per RAD locus.

### Detection of Outlier Loci

Outlier loci that deviated significantly from neutrality were identified with ARLEQUIN 3.5 (Excoffier and Lischer, 2010) using a hierarchical island model where migration rates among groups are different than migration rates among populations within groups. Each species was assigned to a group. Coalescent simulations (50,000) were performed to get the null distribution of F-statistics. The observed data from each locus were compared with the simulated null distribution, and only loci detected as outliers at the significance level of 0.01 were reported as potentially under the effect of selection.

### Genetic Differentiation and Admixture of the Two Species

Genetic differentiation and admixture were assessed by using only SNPs that showed no indication of outlier behavior in AELEQUIN analysis with a model-based Bayesian clustering method implemented in the program STRUCTURE version 2.3.4 (Prichard et al., 2000). Sampling locations were defined as populations in the STRUCTURE analyses. The analyses were conducted by selecting admixture model and correlated allele frequencies between populations. Twenty replications were carried out for each possible cluster (K) being tested from 1 to six, and the length of burn-in and Markov chain Monte Carlo were set to 50,000 and 100,000, respectively. The results were summarized with STRUCTURE HARVESTER (Earl and von Holdt, 2012), and the most likely K was selected by inspecting the second order rate of change of *L(K)* between successive K values (Evanno et al., 2005). Analysis of molecular variance was conducted with ARLEQUIN 3.5 (Excoffier and Lischer, 2010) to partition genetic variance at three levels: species, populations within species, and individuals within populations.

### Two-Dimensional Allele Frequency Spectrum and Alternative Models of Divergence

**Figure 1:**
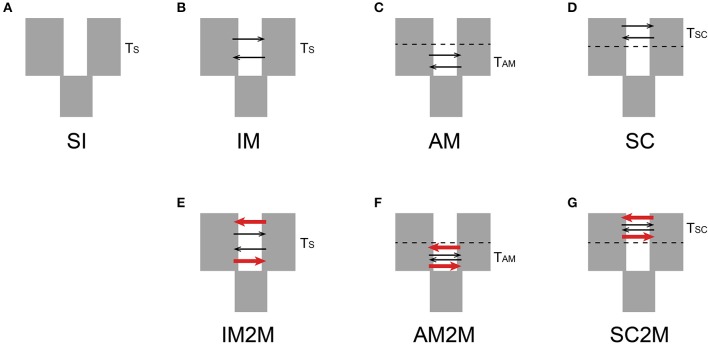
Seven divergence scenarios between *C. fabri* and *C. lamontii* tested in this study. **(A)** SI is the strict isolation model, **(B)** IM is the Isolation-with-Migration model, **(C)** AM is the Ancient Migration model, and **(D)** SC is the Secondary Contact model. Migration assumes to occur with asymmetrical rates in three models (IM, AM, SC). The models of IM, AM, and the SC were extended to **(E)** IM2M, **(F)** AM2M, and **(G)** SC2M, in which two categories of loci with different migration rates were included. T_S_, T_AM_, and T_SC_ represent time of population split, the beginning divergence, and the most recent contact, respectively. The two different arrows represent two categories of loci with different migration rate.

The joint allele frequency spectrum (JAFS) between two species were converted from variant call format (VCF) file using script easySFS (https://github.com/isaacovercast/easySFS). *C. mollissima* was used as outgroup species to determine ancestral vs. derived allelic states for each SNP in order to generate an unfolded JAFS. To account for missing data, the size of the unfolded JAFS was projected down to 32 sampled chromosomes from *C. fabri* and 16 chromosomes from *C. lamontii*. Seven divergence scenarios (Figure 1) between *C. fabri* and *C. lamontii* were tested using δaδi (Gutenkunst et al., 2009). The strict isolation (SI) model corresponds to an allopatric divergence scenario in which the ancestral population splits into *C. fabri* and *C. lamontii* without exchanging genes for Ts generations. The isolation with migration (IM) model refers to evolutionary scenario in which the ancestral population splits into *C. fabri* and *C. lamontii* with gene flow for T_S_ generations. The ancient migration (AM) model and the secondary contact (SC) model assume isolation with gene flow occurring at the beginning of divergence for T_AM_ generations, or at the most recent contact since T_SC_ generation. Migration is assumed to occur with asymmetrical rates (m12 and m21) in all three models (IM, AM, SC). By incorporating the effect of selection, the models of IM, AM, and SC were extended to IM2M, AM2M, and SC2M, in which two categories of loci with heterogenous migration rates, occurring in proportions P and 1-P, were considered (Souissi et al., 2018). Three rounds of optimizations were performed by using the Nelder-Mead simplex algorithm as in a pipeline described in Portik et al. (2017), each round with 100 replicates and a maximum of 100 iterations. Initial optimizations were performed by randomly generating three-fold perturbed parameters, then the best scoring replicate was selected as starting parameters for a second round of two-fold parameter perturbations, and parameter values from the best replicate were subsequently used as one-fold perturbed starting parameters. Evaluation of the frequency spectrum was performed with three progressive grid sizes of 50, 60, and 70. The multinomial approach was used to estimate log-likelihoods of model fit, and the models were compared using the Akaike information criterion (AIC) based on log-likelihood of the highest scoring replicate. The log-likelihood values returned represent the true likelihood values rather than composite likelihoods since only a single SNP was kept per RAD locus (Portik et al., 2017). Parameters were not transformed into biologically meaningful estimates since our primary aim was to perform model selection.

### Climatic Niche Analysis

Climatic variables across the entire distribution range of the two species in subtropical China were obtained with a resolution of 2.5 arcminute through 19 bioclimatic layers (Hijmans et al., 2005). The collection points of the species were obtained from three main herbaria in China (IBSC, KUN, and PE). Ecological niche models were constructed for each species using MAXENT v3.3.3 (Phillips et al., 2006). Model performance was assessed with the mean Area Under the Curve (AUC) of a receiver operating characteristic plot (Fielding and Bell, 1997) across 10 replicates. A mean AUC value >0.7 was considered as evidence that the model had sufficient discriminatory ability (Swets, 1988). Since strong collinearity between environmental variables may bias subsequent analysis (Dormann et al., 2013), R package “MaxentVariableSelection” (Jueterbock et al., 2016) was used to identify the most important set of uncorrelated environmental variables with a contribution threshold of 5% and correlation threshold of 0.70. Seven environmental variables (including BIO2 = Mean Monthly Temperature Range; BIO3 = Isothermality; BIO4 = Temperature Seasonality; BIO6 = Min Temperature of Coldest Month; BIO14 = Precipitation of Driest month; BIO18 = Precipitation of Warmest Quarter; BIO19 = Precipitation of Coldest Quarter) were finally kept in further analysis. Based on the seven climatic variables, the environmental space representing the climatic niche was defined by principal component analysis-env ordination approach (Broennimann et al., 2012) implemented in R package. A kernel density function was used to convert population occurrences into smooth densities in the gridded environmental space. Niche overlap was quantified by Schoener's D metric (Schoener, 1970), and statistical tests of niche equivalency and similarity were performed with 100 permutations by comparing observed *D*-values to simulated overlap distribution.

## Results

**Figure 2:**
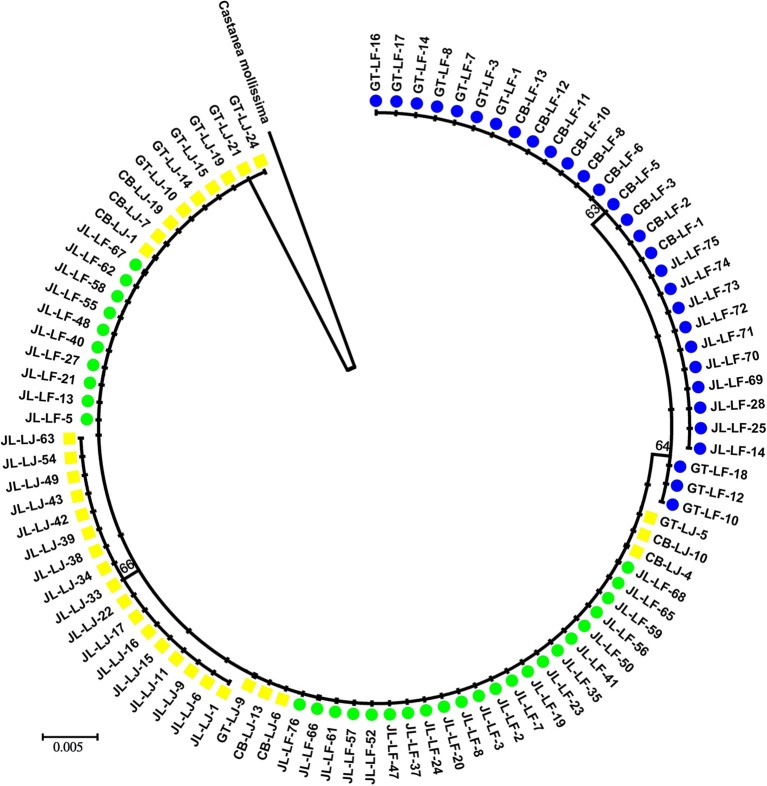
A neighbor-joining tree constructed with chloroplast DNA sequence. Yellow squares are *C. lamontii*. Green circles and blue circles are individuals of *C. fabri* with or without chloroplast exchange, respectively. Numbers at the nodes represent bootstrap values.

**Figure 3:**
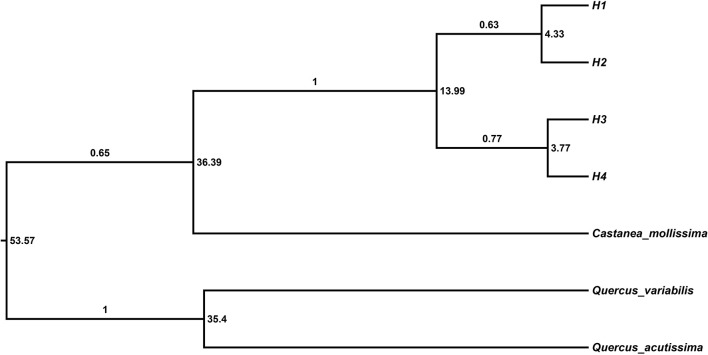
BEAST-derived chronograms for four chloroplast haplotypes. *Castanea mollissima, Querucs variabilis*, and *Quercus acutissima* were used as outgroups. Numbers above braches are posterior probabilities, and divergence times (in Ma) are shown at the right of nodes. Haplotype codes are same as that in Table 1.

After alignment using *C. mollissima* as outgroup, the total length of chloroplast DNA were 1,176 bp, in which 73 sites were variable with alignment gaps considered. Ten sites were variable within the *C. fabri*/*C. lamontii* lineage. Two main clades, generally corresponding to *C. fabri* and *C. lamontii*, were revealed on the neighbor-joining tree (Figure 2) constructed from chloroplast DNA. One clade was composed of 30 individuals of *C. fabri*, including 10 samples from GT-LF, 10 from CB-LF, and 10 from JL-LF. Another clade consisted of the remaining 32 individuals of *C. fabri* from JL-LF and all 32 individuals of *C. lamontii* from GT-LJ, CB-LJ, and JL-LJ. This suggests that the 32 individuals of *C. fabri* from JL-LF have highly similar chloroplast genome with *C. lamontii*. Four chloroplast haplotypes (H1-H4) were distinguished in this study (Table 1). H1 presented in *C. lamontii* from JL-LJ, H2 distributed in C. *lamontii* from CB-LJ and GT-LJ plus 32 individuals of *C. fabri* from JL-LF. H3 was found in three individuals of *C. fabri* from GT-LF, and H4 in the other individuals of *C. fabri* from GT-LF, CB-LF, and JL-LF. Haplotype H1 had a close relationship with H2, while H3 had a close relationship with H4 (Figure 3). Eight nucleotide positions varied between H1 and H2, while only a single substitution differentiated H3 and H4. The time to the most recent common ancestor of the four haplotypes was dated to 13.99 Ma (95% HPD: 1.54–36.8 Ma). Divergence times between H1 and H2 were dated to 4.33 Ma (95%HPD: 0–18.8 Ma), while H3 and H4 separated 3.77 Ma (95%HPD: 0.01–17.15 Ma) ago.

RAD-sequencing produced 602,439–4,774,907 raw reads per individual. The number of calculated RAD clusters with coverage 5 × – 1,000 × was from 39,046 to 272,624 in different individuals, with a median depth of sequencing coverage from 9 to 17. A total of 2,021 RAD clusters were retained for variant calling and generating JASF to test alternative divergence models.

**Figure 4:**
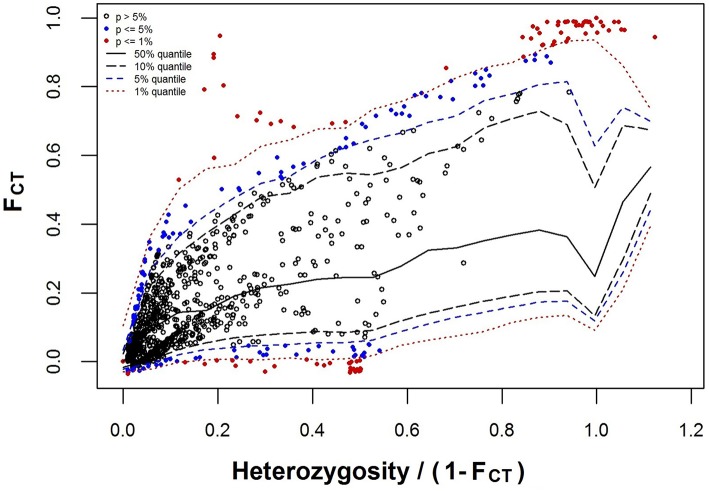
Outlier loci detected with hierarchical island model complemented in ARLEQUIN software. Locus shown as red filled circle was classified as significant outlier as it lay outside of the 99% confidence envelope.

**Figure 5:**
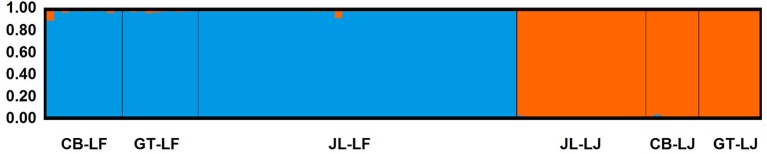
Genetic differentiation of *C. fabri* and *C. lamontii*, and individual proportion of the membership for the inferred clusters defined by the STRUCTURE analysis. All individuals had >0.91 assignment probability to their respective populations. Population codes are same as that in Table 1.

At the significance level of 0.01, a total of 112 loci were detected as outliers (Figure 4), in which 84 Loci that presented F_CT_ higher than the 99% confidence interval were considered candidate for divergent selection, and 28 loci that presented F_CT_ lower than the 99% confidence interval were considered candidate for balancing selection. Hierarchical analysis of molecular variance of 1,909 neutral SNPs revealed that the majority of molecular variation (71.21%) was distributed within populations, 6.97% among populations within species, and 21.82% among species (FST = 0.288, *P* = 0.000; FSC = 0.089, *P* = 0.000; FCT = 0.218, *P* = 0.093). In STRUCTURE analyses, an optimal *K* = 2 was obtained from the plot of ΔK against a range of K values (from 1 to 6). Two distinct gene pools identified were corresponding well to the two species, suggesting clear genetic differentiation between *C. fabri* and *C. lamontii* (Figure 5). Little signal of genetic admixture between the two species was revealed in the nuclear genome, since the estimate of membership coefficient (*Q*-value) exceeded 0.91 in all individuals for either of the two main clusters.

**Figure 6:**
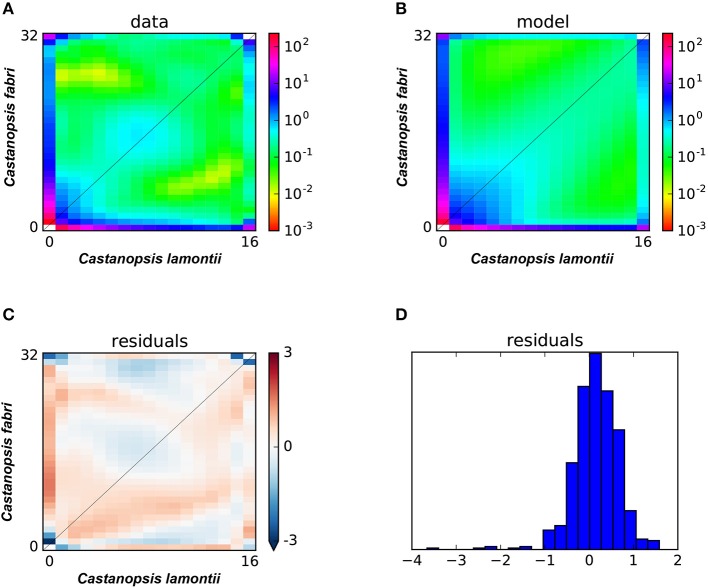
**(A)** Observed two-dimensional site frequency spectra (2D-SFS) for *C. fabri* and *C. lamontii*; **(B)** the expected 2D-SFS for *C. fabri* and *C. lamontii* generated from SC2M model; **(C,D)** the residuals for fitting expected spectrum to observed spectrum.

In statistical tests with δaδi, the model of secondary contact with varying gene exchange rates across the genome (SC2M) provided the best fit to the observed JAFS (Figure 6) compared with the other six alternative models, indicating that this scenario best explained the evolutionary history of the two species. These results suggested that *C. fabri* and *C. lamontii* accumulated genomic differences during a period of isolation, but subsequently exchanged genes during secondary contact. Gene exchange during secondary contact was strongly asymmetrical, mainly from *C. lamontii* to *C. fabri*, and occurred at highly variable rates across the genome (Table 2). It is estimated that ~73% of the genome did not freely exchange from one species to the other, while the other proportion of the genome exchange neutrally.

**Table 2:** Demographic models and parameter values (unscaled) for divergence between *Castanopsis fabri* and *Castanopsis lamontii*, the best fit model is a secondary contact scenario with heterogeneous introgression rates among loci.

**Model**	**Log-likelihood**	**AIC**	**theta**	**N1**	**N2**	**m12**	**m21**	**me12**	**me21**	**T_**S**_**	**T_**AM**_**	**T_**SC**_**	***P***
SC2M	−383.26	784.52	94.03	1.7785	1.6024	4.1314	0.0438	0.0264	0.0331	1.7599	–	0.714	0.3721
IM2M	−385.49	786.98	87.27	2.0352	1.6682	2.9168	0.0119	0.0166	0.0326	2.9395	–	–	0.4183
AM2M	−387.98	793.96	119.9	1.4719	1.1643	0.0222	0.0488	4.1624	0.044	1.9306	0.0155	–	0.5701
SC	−442.93	897.86	121.29	1.5987	1.1098	0.1291	0.2257	–	–	0.9562	–	0.1193	–
IM	−444.65	899.3	112.59	1.7058	1.1	0.0514	0.1519	–	–	1.3738	–	–	–
AM	−444.65	901.3	112.56	1.7083	1.1	0.0512	0.1514	–	–	1.372	0.0	–	–
SI	−482.87	971.74	145.92	1.3593	0.9246	–	–	–	–	0.6686	–	–	–

**Figure 7:**
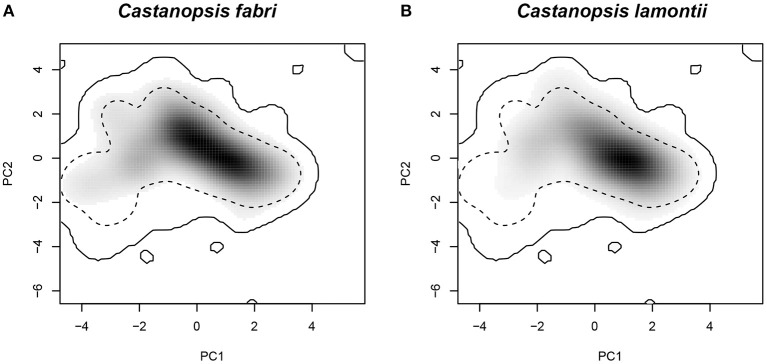
Climatic niche quantification based on principal component analysis-env ordination approach. **(A)** Niche occupied by *C. fabri*; **(B)** niche occupied by *C. lamontii*. The solid and dashed contour lines illustrate 100 and 50% of available environment.

The two species occupied roughly similar parts of environmental space (Figure 7); however, *C. fabri* showed more occurrence densities in the upper left quadrant, which was primarily aligned with the minimum temperature of coldest month and the precipitation of warmest Quarter. The value of niche overlap (D) between *C. fabri* and *C. lamontii* was 0.734. Niche equivalency hypothesis was not rejected (*P* = 0.5), indicating that the two species occupied equivalent parts of environment space. Niche similarity hypothesis was significant (*P* < 0.05), suggesting that the climatic niches of the two species were more similar than would be expected by chance.

## Discussion

### Interspecific Gene Exchange

The evidence of gene exchange between *C. fabri* and *C. lamontii* comes from the fact that some individuals of *C. fabri* had similar chloroplast genome of *C. lamontii*, which is a consequence of chloroplast genome capture of *C. lamontii* by *C. fabri* through hybridization and introgression. It is not very probable that the intertwined pattern of *C. fabri* and *C. lamontii* on the chloroplast gene tree is due to incomplete lineage sorting, since the time to the most recent common ancestor of the four haplotypes was dated to quite a long time (13.99 Ma) ago, and the shared haplotype H2 was well-diverged from the other haplotypes (H3 and H4) found in *C. fabri*. In addition, natural hybridization and introgression among *Castanopsis* species has been documented between *C. tibetana* Hance and *C. sclerophylla* (Lindl.) Schott., and between *C. eyrei* (Champ.) Tutch. and *C. lamontii* Hance (Huang and Chang, 1998).

Given the maternal inheritance of chloroplast genome, some individuals of *C. fabri* having more similar chloroplast genome to that of *C. lamontii* implies that the initial hybridization happens between *C. lamontii* as pistillate and *C. fabri* as staminate parent. Hybridization and introgression between *C. lamontii* and *C. fabri* could be attributed to an overlap in flowering phenology of the two species. *C. lamontii* is usually flowering from March to May, while *C. fabri* is flowering in April to May (Huang and Chang, 1998). Introgression seems to be unidirectional, which is reasonable in light of the fact that *C. lamontii* inhabits lower elevations and would have a greater opportunity of receiving pollen from *C. fabri*. The possibilities of interspecific crossing may increase more if we consider that *Castanospsis* species has high potential for long-distance dispersal of pollen (Nakanishi et al., 2012) and the related oak trees are generally protandrous (Belahbib et al., 2001).

On the other hand, species relative abundance has an important impact on the direction of introgression, which is predominantly toward the more numerous species (Lepais et al., 2009). The evergreen broad-leaved forest at Jiulianshan is in the phase of succession toward the stable stage, in which *C. fabri* has higher frequency than *C. lamontii* (Hideyuki et al., 2005; Jian et al., 2009). When the less abundant *C. lamontii* receive many hetero-specific pollens from the more frequent *C. fabri*, the chances of mate-recognition errors will increase and thus hybridization occurs. Moreover, first-generation hybrids will also more likely backcross with the more abundant species, leading to asymmetrical introgression (Lepais et al., 2009). The asymmetrical introgression is supported by the best-fit divergence model, where migration rates from *C. lamontii* to *C. fabri* at neutral loci are much higher than in the opposite direction (m12 >> m21, Table 2).

The individual-based genetic clustering revealed distinct nuclear genome differentiation between *C. fabri* and *C. lamontii*, and the estimate of membership coefficient (*Q*-value) exceed 0.91 in all individuals for either of the two main genetic clusters. This is generally in conformity with observation of more extensive cpDNA introgression than nrDNA introgression is revealed in plant genera, which usually attributed to the haploid nature and uniparental inheritance of cpDNA, less effective seed dispersal than pollen dispersal, and negative selection acting on nrDNA introgression (Rieseberg et al., 1991; Martinsen et al., 2001; Mckinnon et al., 2010).

### Divergence History of the Two Species

Clearly genetic differentiation between *C. fabri* and *C. lamontii* is revealed by RAD-sequencing data (Figure 5). In terms of the demographic history of divergence between *C. fabri* and *C. lamontii*, the model of allopatric isolation followed by recent secondary contact is best-fitted to our data. Repeated glaciation cycles could push the tree species down/up the hill slope in mountain areas of subtropical China (Yan et al., 2012), and lead to the two species mixing in a forest as our case. Coinciding with the obvious climatic shifts in region of eastern China during the middle Pleistocene, demographic contraction followed by expansion of *Castanopsis* species has been detected in this region (Sun et al., 2014), which strengthen the inference of the secondary contact scenario between *C. fabri* and *C. lamontii*.

The gene exchange between the two species generates a very heterogeneous distribution of gene flow rates throughout the genome, and a divergence model including two categories of loci with different migration rate obtains the highest support in our study. It is assumed that selection contributes to heterogeneous gene exchange rate across the genome (Martinsen et al., 2001), and one important line of evidence comes from our results that 112 outlier loci potentially under the effect of selection were detected in this study. In our case, the chloroplast exchange between *C. fabri* and *C. lamontii* is not seen in populations Gutian and Chebaling where the two species occupy different elevations on hill slope, indicating the existence of reproductive barriers among the two species due to environmental selection or post-divergence selection (Filatov et al., 2016). The chloroplast exchange between *C. fabri* and *C. lamontii* is discovered in Jiulianshan forest stand where the two species have a recent contact, suggesting that the two *Castanopsis* species connect by episodic gene flow after secondary contact, as discovered in European white oak species (Leroy et al., 2017).

Gene exchange is promoted by niche overlap when the two species show niche conservatism (Wu et al., 2015), and may play a potential role to widen a species' niche (Choler et al., 2004). Substantial niche overlap is found between *C. fabri* and *C. lamontii*, and the similarity test indicates the niches of the two species are more similar than would be expected at random. *C. fabri* and *C. lamontii* show positive association at Jiulianshan forest, although not significant, highlighting that the two species show similarity of resource utilization and the overlap of niches (Jian et al., 2009; Xu and Cai, 2016). Gene exchange allows *C. fabri* to widen its habitat into low elevation through pollen swamping, exactly as a process described for sympatric European oak species (*Quercus petraea* and *Q. robur*) (Petit et al., 2003). During this process, the chloroplast genome of *C. lamontii* is captured by *C. fabri*, and the latter species broadens its climatic niches to be more tolerant to harsh conditions, such as the minimum temperature in coldest month and the precipitation in warmest season.

## Conclusions

Our study illustrates clear genetic differentiation between *C. fabri* and *C. lamontii*, and depicts signatures of gene exchange between the two species as a consequence of secondary contact. Specifically, we inferred unidirectional chloroplast introgression and the variable gene exchange rates across the genome. The findings of this study can help understand how related species have maintained their species integrity in the face of gene flow.

## Data Availability Statement

Chloroplast DNA sequences have been deposited in GenBank (Accession Nos. MF592798-MF592985). Raw Illumina sequences of RAD-seq have been deposited in China National GeneBank (https://db.cngb.org/cnsa), CNGB Project ID CNP0000496.

## Author Contributions

YS and XW conceived and designed the experiments and wrote the paper. YS performed experiments and analyzed the data.

### Conflict of Interest

The authors declare that the research was conducted in the absence of any commercial or financial relationships that could be construed as a potential conflict of interest.
